# Oligodendrocyte Lineage Marker Expression in eGFP-GFAP Transgenic Mice

**DOI:** 10.1007/s12031-020-01771-w

**Published:** 2020-12-21

**Authors:** Newshan Behrangi, Peter Lorenz, Markus Kipp

**Affiliations:** 1 Institute of Anatomy, Rostock University Medical Center, 18057 Rostock, Germany; 2 Department of Anatomy II, Ludwig-Maximilians-University of Munich, 80336 Munich, Germany; 3 Institute of Immunology, Rostock University Medical Center, 18057 Rostock, Germany; 4 Center for Transdisciplinary Neurosciences Rostock (CTNR), Rostock University Medical Center, Gelsheimer Strasse 20, 18147 Rostock, Germany

**Keywords:** APC-CC1, OLIG2, NG2, eGFP-GFAP

## Abstract

Oligodendrocytes, the myelinating cells of the central nervous system, orchestrate several key cellular functions in the brain and spinal cord, including axon insulation, energy transfer to neurons, and, eventually, modulation of immune responses. There is growing interest for obtaining reliable markers that can specifically label oligodendroglia and their progeny. In many studies, anti-CC1 antibodies, presumably recognizing the protein adenomatous polyposis coli (APC), are used to label mature, myelinating oligodendrocytes. However, it has been discussed whether anti-CC1 antibodies could recognize as well, under pathological conditions, other cell populations, particularly astrocytes. In this study, we used transgenic mice in which astrocytes are labeled by the enhanced green fluorescent protein (eGFP) under the control of the human glial fibrillary acidic protein (GFAP) promoter. By detailed co-localization studies we were able to demonstrate that a significant proportion of eGFP-expressing cells co-express markers of the oligodendrocyte lineage, such as the transcription factor Oligodendrocyte Transcription Factor 2 (OLIG2); the NG2 proteoglycan, also known as chrondroitin sulfate proteoglycan 4 (CSPG4); or APC. The current finding that the GFAP promoter drives transgene expression in cells of the oligodendrocyte lineage should be considered when interpreting results from co-localization studies.

## Introduction

The adult vertebrate central nervous system (CNS) mainly consists of neurons, astrocytes, oligodendrocytes, and microglia cells. Oligodendrocytes, the myelin forming cells of the CNS, are subjected to cell stress or even death in a number of metabolic or inflammatory disorders, among multiple sclerosis (MS) (Armati and Mathey 2010; Bradl and Lassmann 2010; Caprariello et al. 2012; Jäkel and Dimou 2017; Traka et al. 2016). MS is associated with the development of large demyelinated plaques, oligodendrocyte destruction, and axonal degeneration (DeLuca et al. 2006; Popescu et al. 2013). This pathology is paralleled by the activation of astrocytes and microglia as well as the recruitment of peripheral immune cells to the site of tissue injury. Underlying mechanisms of demyelination and neurodegeneration in MS are still poorly understood.

Any pathogenetic concept of MS has to provide an explanation for the highly specific destruction of myelin and oligodendrocytes. Although the sequence of molecular events leading to oligodendrocyte loss and consequently demyelination are not fully understood, different stressors are known which can induce oligodendrocyte degeneration including oxidative stress, mitochondrial dysfunction, nitric oxide, protein misfolding, or inflammatory cytokine exposure (Aboul-Enein and Lassmann 2005; Fischbach et al. 2019; Smith and Lassmann 2002). Of note, viable oligodendrocytes and an intact myelin sheath are indispensable for neuronal health. For example, it has been shown that oligodendrocytes provide nutritional support to neurons (Funfschilling et al. 2012), that fast axonal transport depends on oligodendrocytes (Edgar et al. 2004), and that mice deficient for mature myelin proteins display severe neurodegeneration (Uschkureit et al. 2000).

To study oligodendrocyte development, physiology and pathology, their reliable visualization is indispensable. Numerous antibodies for this purpose exist, among anti-transcription factor Oligodendrocyte Transcription Factor 2 (OLIG2) (Draheim et al. 2016; Leopold et al. 2019; Scheld et al. 2019) antibodies, anti-NG2 proteoglycan antibodies, also known as chrondroitin sulfate proteoglycan 4 (CSPG4), or the monoclonal antibody anti‐adenomatous polyposis coli (APC) clone CC1, hereafter referred to as the CC1 antibody (Bin et al. 2016). Few markers exist that specifically label mature oligodendrocyte cell bodies without labeling the surrounding myelin. Thus, when staining for oligodendrocytes, the cell bodies typically are masked by the abundant surrounding myelin, making them difficult to identify or precisely count. CC1 antibodies are the most commonly used tool to specifically visualize mature oligodendrocytes without labeling myelin under different experimental conditions including brain ischemia (Ahrendsen et al. 2016), spinal cord injury (Duncan et al. 2018), Alzheimer´s disease (Quintela-López et al. 2019) and, most importantly, in models mimicking aspects of MS (Kirby et al. 2019; Krauspe et al. 2015; Namekata et al. 2014; Stone et al. 2017).

Adenomatous polyposis coli (APC) is a tumor suppressor regulating cell differentiation via the Wnt pathway (Näthke 2004; Segditsas and Tomlinson 2006). In the brain, it has been suggested that APC is not just expressed by mature oligodendrocytes (Bhat et al. 1996), but can as well be expressed by astrocytes (Etienne-Manneville and Hall 2001, 2003) or astrocyte subpopulations such as Bergmann glia (Bhat et al. 1996). In contrast to that view, it has been shown in the cuprizone model of demyelination (Kipp et al. 2009; Praet et al. 2014) that APC co-localizes with the oligodendrocyte marker protein NogoA but does not co-localize with the astrocytic marker GFAP (Salinas Tejedor et al. 2015), suggesting that at least in this model of metabolic-oligodendrocyte degeneration, CC1 antibodies do not label astrocytes.

Based on these contradictory findings, in this study we used GFAP promoter-controlled eGFP-expressing transgenic mice as a tool to visualize astrocytes to answer the question whether or not CC1 antibodies label astrocytes in the control brain and after cuprizone-induced toxic demyelination (Nolte et al. 2001).

## Material and Methods

### Cuprizone Intoxication in Mice

Experiments were either performed with C57BL/6 female mice (19–20 g) which were purchased from Janvier Labs, Le Genest-Saint-Isle, France or with GFAP-eGFP transgenic mice which were bred and maintained in our own animal facility (Nolte et al. 2001). These latter mice express eGFP under the control of the human GFAP promotor. Genomic DNAs obtained from tail biopsies were tested by PCR using the following primers: eGFP gene (GenBank accession number U55761, from sequence of cloning vector peGFP-1) 5′-TCGAGCTGGACGGCGACGTAAA-3′(sense) and 5′-TAGTGGTTGTCGGGCAGCAGCA-3′(antisense). All mice were housed and monitored consistent with the Federation of European Laboratory Animal Science Associations recommendations. They were maintained in a temperature-controlled environment (21–24 °C) and humidity levels between 55 and 65% on a 12-h light/dark cycle. Red houses (Tecniplast, Hohenpeißenberg, Germany) and nesting materials (Nestlets; Ssniff, Soest, Germany) were provided, and food and water were available ad libitum. All experimental procedures were approved by the Review Board for the Care of Animal Subjects of the district government of Upper Bavaria (55.2-1-54-2532-73-15). To induce acute demyelination, 9-week-old (24–25 g) mice were fed a diet containing 0.25% cuprizone (Sigma- Aldrich, Taufkirchen, Germany) mixed into a ground standard rodent chow (V1530-0; Ssniff) for 5 weeks. Age matched control mice (*n* = 9) were fed throughout the experimental procedure with cuprizone-free standard rodent chow. Chow containing 0.25% cuprizone was freshly prepared every day as follows: 0.25 g cuprizone was weighed using precision scales and mechanically mixed with 100 g ground standard rodent chow using a commercial available kitchen machine (Kult X, WMF Group, Geislingen an der Steige, Germany). The chow was mixed at low speed and manual agitation for 1 min and was provided within the cage in two separate plastic petri dishes.

### Tissue Preparation

Preparation of tissues was performed as previously described (Acs et al. 2009; Groebe et al. 2009). In brief, mice were transcardially perfused with ice-cold phosphate buffered saline (PBS) followed by 4% paraformaldehyde (pH 7.4). After overnight postfixation in the same fixative, some brains were dissected and post-fixed in PBS overnight for cryo embedding, followed by sequential equilibration in 10%, 20%, and finally 30% sucrose-containing PBS. Brains were quickly frozen in isopentane cooled by dry ice. The other brains were embedded in paraffin. Then, either cryo sections of 15-μm or paraffin sections of 5-μm thickness were prepared for immunohistological investigations. Brains were sectioned at the level 265 according to the mouse brain atlas by Sidman et al.(http://www.hms.harvard.edu/research/brain/atlas.html). Region 265 corresponds to the stereotaxic coordinates Bregma-1.01, provided by Franklin and Paxinos.

### Histology and Immunohistochemistry

For histochemical evaluations, randomly selected brain sections containing the region of interest (ROI) were stained with standard Luxol fast blue-periodic acid-Schiff (LFB/PAS) to determine demyelination and hypercellularity. For immunofluorescence double staining, cryo-sections were washed with PBS and incubated in blocking solution containing 5% donkey serum and 0.5%Triton solution in PBS for 1 h at room temperature. Sections were then incubated in a combination of the following primary antibodies for 24 h at 4 °C diluted in blocking solution: anti-adenomatous polyposis coli gene clone CC1 (APC/CC1), anti-oligodendrocyte transcription factor (OLIG2), anti-neuron glial antigen-2 (NG2), or anti-glial fibrillary acidic protein (GFAP) (see Table 1). After washing, sections were incubated for 1 h at room temperature with fluorescent secondary antibodies (see Table 2) diluted in blocking solution. Sections were then incubated in 4′, 6-diamidino-2-phenylindole (DAPI) (Roth, Germany; 1:5000) solution diluted in distilled water for the staining of cell nuclei. Unspecific secondary antibody bindings to the tissue itself were excluded by incubating sections with the fluorescent secondary antibodies alone.

**Table 1:** List of primary antibodies used in this study

Antigen	Host/clone	Dilution	Purchase number	RRID	Supplier
PLP	Mouse monoclonal	1:5000	MCA839G	AB_2237198	Biorad, USA
IBA1	Rabbit Polyclonal	1:5000	019-19741	AB_839504	WAKO, USA
APC /CC1	Mouse monoclonal	1:100 (Fluorescence)	OP80	AB_2057371	Millipore, USA
OLIG2	Rabbit Polyclonal	1:1000 (Fluorescence)	AB9610	AB_570666	Millipore, USA
NG2	Rabbit Polyclonal	1:50 (Fluorescence)	AB5320	AB_11213678	Millipore, USA
GFAP	Chicken Polyclonal	1:4000 (Fluorescence)	AB4674	AB_304558	Abcam, UK

**Table 2:** List of secondary antibodies used in this study

Antigen	Host/clone	Dilution	Purchase number	RRID	Supplier
Goat anti-rabbit IgG	Goat Polyclonal	1:200	BA-1000	AB_2313606	Vector Laboratories, Burlingame, USA
Goat anti-mouse IgG	Goat Polyclonal	1:200	BA-9200	AB_2336171	Vector Laboratories, Burlingame, USA
Donkey anti-rabbit IgG (H + L) Highly, cross-adsorbed secondary antibody, Alexa Fluor 594	Donkey Polyclonal	1:200	A21207	AB_141637	Thermo Fisher Scientific, USA
Donkey anti-mouse IgG (H + L) Highly cross-adsorbed secondary antibody, Alexa Fluor 594	Donkey Polyclonal	1:200	A21203	AB_141633	Thermo Fisher Scientific, USA
Donkey anti-chicken IgG (H + L) Highly cross-adsorbed secondary antibody, Alexa Fluor 488	Donkey Polyclonal	1:200	A11039	AB_142924	Thermo Fisher Scientific, USA
Alexa Fluor 647-AffiniPure Goat Anti-Rabbit IgG (H + L) (min X Hu, Ms, Rat Sr Prot)	Goat Polyclonal	1:200	111-605-144	AB_2338078	Jackson Immuno Research, USA

For immunohistochemistry, paraffin sections were deparaffinized, rehydrated, if necessary heat-unmasked in either citrate (pH 6.0) or Tris/EDTA-buffer (pH 9.0), and blocked with PBS containing 5% normal serum or a mixture of 2% normal serum, 0.1% cold water fish skin gelatin, 1% bovine serum albumin, and 0.05% Tween-20. Thereafter, slides were incubated overnight at 4 °C with the following primary antibodies diluted in blocking solution: anti-Proteolipid protein (PLP) or anti- ionized calcium-binding adaptor molecule 1 (IBA1). Sections were subsequently incubated with biotinylated secondary antibodies for 1 h at room temperature, followed by peroxidase-coupled avidin–biotin-complex (ABC kit; Vector Laboratories). The antigen-antibody conjugate was then visualized with 3,3′-diaminobenzidine (DAB; DAKO). Sections were counterstained with standard hematoxylin to visualize cell nuclei, if appropriate.

### Histological Evaluations

Stained and processed sections were digitalized using a Leica DM6 B microscope equipped with a Leica DMC 6200 camera or the confocal laser scanning microscope Leica TCS SP2 AOBS (Version 07.01.2020 Lo) (Wetzlar, Germany). Low-magnification images were used to perform the densitometry analyses. High-magnification images were used for all quantitative analyses. The software packages LAS-X-Leica Microsystem CMS-GmbH Version 3.70.20979, LAS AF Lite 2.6. 7266, or Image J software were used to quantify the co-localization of glial cell markers in the region of interest. To estimate demyelination in LFB/PAS stained sections, a nonparametric grading approach was performed by a blinded evaluator (N.B.) from 1 (complete demyelination) to 4 (fully myelinated) within the midline of the corpus callosum (CC) at the brain level R265. To quantify the relative expression of anti-CC1, anti-OLIG2, anti-NG2, or anti-GFAP in the eGFP^+^ cells, an evaluator blinded to the treatment group (N.B.) first identified the medial CC, the lateral CC, and the primary motor and somatosensory cortex (CX) areas. In a next step, all eGFP^+^ cells were identified and then, in a third step, it was evaluated which of the marked eGFP^+^ cells were as well labeled by the respective applied antibodies. The results are, therefore, shown as percentage of eGFP-GFAP^+^ cells expressing either CC1^+^, OLIG2^+^, NG2^+^, or GFAP^+^, respectively.

### Statistical Analyses

Differences between groups were statistically tested using GraphPad Prism 5. In all cases, the Kolmogorov-Smirnov test was applied to test for normal data distribution. To compare two groups, the Mann-Whitney *U* test was used for non-parametric data. All data are given as arithmetic means ± standard error of the mean (SEM). Significance levels are indicated as *p < 0.05, **p < 0.01, and ***p < 0.001.

## Results

### Cuprizone Induces Demyelination and Glia Cell Activation in Wild-Type and eGFP-GFAP Mice

**Fig. 1:**
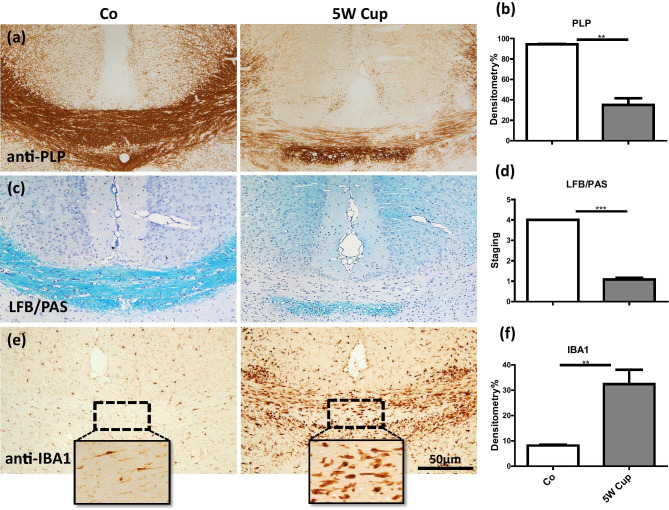
Immunohistochemical analysis of demyelination and microglia/macrophage activation after cuprizone intoxication for 5 weeks. **(a)** Representative anti-PLP stained sections from a control and 5-week cuprizone intoxicated mouse. **(b)** Results of the densitometric evaluation of anti-PLP stained sections. **(c)** Representative LFB/PAS stained sections from a control and 5-week cuprizone intoxicated mouse. **(d)** Evaluation of the extent of demyelination of LFB/PAS stained sections. **(e)** Representative anti-IBA1 stained sections from a control and 5-week cuprizone intoxicated mouse. **(f)** Results of the densitometric evaluation of anti-IBA1 stained sections. In total, five to six animals per group were processed; two independent experiments were performed. Significant differences are indicated by asterisks (*p < 0.05, **p < 0.01, ***p < 0.001). Scale bar = 10 µm

First, we were interested whether cuprizone induces demyelination in eGFP-GFAP mice. To this end, mice were intoxicated with cuprizone for 5 weeks. After week 5, the mice were sacrificed and their brains were analyzed for demyelination and the activation of microglial cells at the level of the rostral hippocampus (R 265 according to the online mouse brain atlas published by Sidman et al. (Sidman et al. 1971)). As shown in Fig. 1a, c, in control animals, the medial corpus callosum (CC) was fully myelinated (anti-PLP and LFB/PAS stains), whereas animals intoxicated with 0.25% cuprizone showed almost complete demyelination. Demyelination was paralleled by the accumulation of IBA1 expressing microglia, which was particularly pronounced in areas of complete demyelination (Fig. 1e). Unbiased evaluation of anti-PLP, LFB/PAS, and anti-IBA1 stained sections revealed a highly significant demyelination and microglia activation, respectively (Fig. 1b, d, f). Comparable toxic effects of cuprizone were observed in C57BL/6 wild-type mice (data not shown).

### Distribution of eGFP and Anti-GFAP^+^ Cells

In this study, astrocytes are visualized by two different methods. Firstly, in eGFP-GFAP transgenic mice where GFAP-expressing cells co-express eGFP, and secondly by anti-GFAP immunohistochemistry. The former cells will be called eGFP^+^ cells, the latter anti-GFAP^+^ cells.

**Fig. 2:**
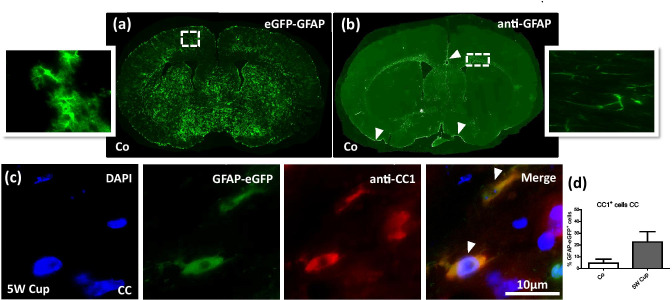
Comparison of eGFP-GFAP expression and anti-GFAP immunostaining in control and after cuprizone intoxication. **(a), (b)** The distribution of eGFP-GFAP^+^ cells and anti-GFAP^+^ cells in the brain of a control mouse (*Co*). Higher magnification images are taken from the cortex and lateral part of the corpus callosum. The star indicates the anterior commissure, arrowheads highlight the brain surface (i.e., glia limitans superficialis). **(c)** Immunofluorescence labeling of anti-CC1 (red) in eGFP-GFAP (green) transgenic mice. In total, three to four animals per group were processed; two independent experiments were performed. Arrowheads indicate co-localization of anti-CC1 and eGFP-GFAP signals. **(d)** The quantification of eGFP-GFAP GFAP$^+$  and anti-CC1$^+$ cells in the corpus callosum (CC). Scale bar = 10 µm

In a first step, we compared the spatial distribution of eGFP^+^ cells in our eGFP-GFAP mice with the spatial distribution of anti-GFAP^+^ cells in wild-type C57BL6 mice. As demonstrated in Fig. 2a, b, eGFP^+^ cells were found to be widely distributed within the entire brain, including the grey matter cortex, corpus callosum, caudate putamen, or hypothalamus. In contrast, densities of anti-GFAP^+^ cells were particularly high in the white matter tracts corpus callosum and anterior commissure (see star in Fig. 2b), in close vicinity to the brain surface (see arrowheads in Fig. 2b) and around blood vessels, whereas other brain areas, such as the cortex and caudate putamen, were just faintly stained by the anti-GFAP antibodies. On the cellular level, anti-GFAP immunostaining mainly visualized the thick primary processes and parts of the cell body, whereas eGFP^+^ cells display the complex morphology of astrocytes, including the cell body, the thick primary processes but equally the finely-branched peripheral processes of astrocytes (see high power views in Fig. 2a, b). This difference in the morphological appearance of the cells most likely is due to the fact that eGFP is a cytoplasmically expressed diffusible protein, while GFAP is a cytoskeletal protein assembled in intermediate filament bundles (Nolte et al. 2001).

### eGFP-GFAP Cells Express Oligodendrocyte Lineage Marker Proteins

It has been suggested that APC is not just expressed by mature oligodendrocytes (Bhat et al. 1996), but can as well be expressed by astrocytes (Sakamoto, Boëda et al. 2013) or astrocyte subpopulations such as Bergmann glia (Bhat et al. 1996). To analyze, whether APC and eGFP co-localize under physiological or stress conditions, we processed brain sections from control and cuprizone-intoxicated eGFP-GFAP transgenic mice for anti-CC1 immunofluorescence staining and quantified the co-localization of both signals. To quantify the relative expression of APC in the eGFP^+^ cell populations, an evaluator (N.B.) blinded to the treatment group first labeled all eGFP^+^ cells and looked in a second step, which of the marked eGFP^+^ cells were labeled by the anti-CC1 antibodies. As demonstrated in Fig. 2c, d, very low numbers of eGFP^+^ cells co-expressed APC in the CC of control mice, whereas around one fourth (~ 22.6%) of eGFP^+^ cells were co-labeled by anti-CC1 antibodies in the CC of cuprizone-intoxicated mice.

**Fig. 3:**
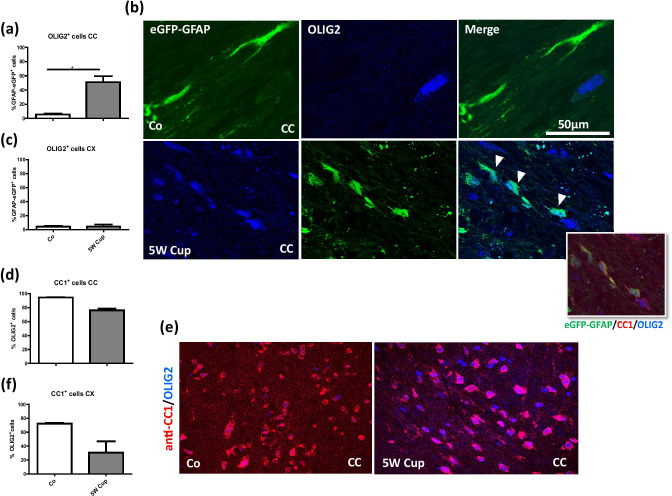
Immunohistochemical analysis of anti-CC1 (red) and anti-OLIG2 (blue) in eGFP-GFAP (green) transgenic mice. **(a, c)** The quantification of eGFP-GFAP^+^ cells expressing OLIG2 or CC1 in the CC and CX, respectively. **(b)** Representative images of eGFP-GFAP brain sections in the corpus callosum (*CC*), processed for anti-CC1 and anti-OLIG2 staining in control and cuprizone-intoxicated mice. Arrowheads indicate the co-localization of eGFP-GFAP and OLIG2. **(d), (f)** The quantification of double positive anti-OLIG2^+^/anti-CC1^+^ cells in the CC and CX, respectively. **e** Representative images of anti-CC1 (red) and anti-OLIG2 (blue) in the midline of the CC. Note that all anti-CC1^+^ cells stained positive for anti-OLIG2. Four animals per group; two independent experiments were performed. Statistically significant differences are indicated by asterisks (*p < 0.05). Scale bar = 50 µm

Surprised by the relatively high percentage of co-expression, we performed a number of control experiments. First, to verify that eGFP^+^ cells co-express markers of the oligodendrocyte lineage, we processed brain sections from control and cuprizone-intoxicated transgenic mice for anti-OLIG2 immunofluorescence staining and quantified the co-localization of the signals following the procedure as described above. As demonstrated in Fig. 3a, very low numbers of eGFP^+^ cells co-expressed OLIG2 in the CC of control mice (5.4%), whereas more than a half (51.1%) of eGFP^+^ cells co-expressed OLIG2 in the CC of cuprizone-intoxicated mice. Of note, an increase of double eGFP^+^/OLIG2^+^ cells under stress conditions was not found in the grey matter cortex (~ 4.5% in control and cuprizone mice; Fig. 3c).

To verify that the applied anti-CC1 antibody reliably detects cells of the oligodendrocyte lineage, we processed brain sections from control and cuprizone-intoxicated wild-type mice for anti-OLIG2/anti-CC1 immunofluorescence double-staining, and quantified the co-localization of both signals. As demonstrated in Fig. 3d, in the CC of control mice, 94.6% of OLIG2^+^ cells were co-labeled with anti-CC1 antibodies, whereas in cuprizone-intoxicated mice, 76.1% of the OLIG2^+^ cell population were co-labeled with anti-CC1 antibodies. Comparably, in the CX of control mice, 72.2% of OLIG2^+^ cells were co-labeled with anti-CC1 antibodies, whereas in cuprizone-intoxicated mice, 30.6% of the OLIG2^+^ cell population was co-labeled with anti-CC1 antibodies. Of note, virtually all anti-CC1 cells expressed the transcription factor OLIG2 (data not shown), clearly demonstrating that the applied antibody reliably detects its antigen. Beyond, anti-CC1/anti-OLIG2 double labeling in eGFP-GFAP mice clearly demonstrated co-localizations after 5-week cuprizone intoxication (see insert in Fig. 3b).

**Fig. 4:**
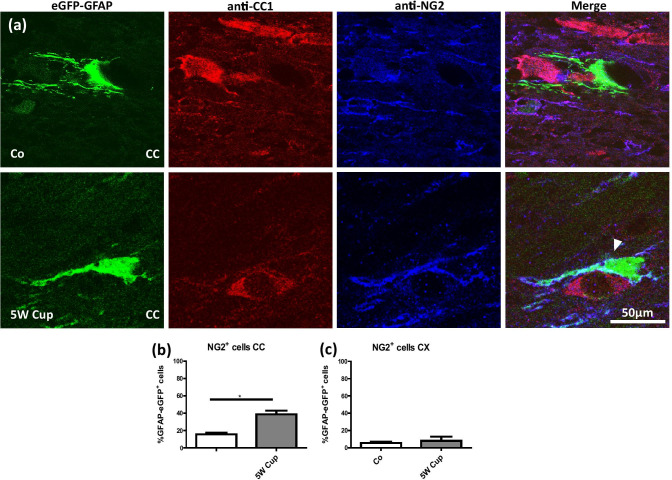
Immunohistochemical analysis of anti-CC1 (red), and anti-NG2 (blue) in eGFP-GFAP (green) mice after cuprizone intoxication. **(a)** Representative sections of eGFP-GFAP, anti-CC1, and anti-NG2 stains in the CC of control (*Co*) and 5-week cuprizone (5 W Cup) intoxicated mice. The white arrowhead highlights the co-localization of eGFP-GFAP and anti-NG2^+^. **(b) (c)** The quantification of eGFP-GFAP/anti-NG2 co-localization in the CC and CX, respectively. Four animals per groups; two independent experiments. Statistically significant differences are indicated by asterisks (*p < 0.05). Scale bar = 50 µm

To further strengthen the observation that eGFP^+^ cells co-express markers of the oligodendrocyte lineage, we processed brain sections from control and cuprizone-intoxicated transgenic mice for anti-NG2/anti-CC1 immunofluorescence double staining and quantified the co-localization of the signals. As demonstrated in Fig. 4a, b, 15.6% of eGFP^+^ cells co-expressed NG2 in the CC of control mice, whereas 38.8% of eGFP^+^ cells co-express NG2 in the CC of cuprizone-intoxicated mice. No increase in co-labeling was observed in the cortex (Fig. 4c).

**Fig. 5:**
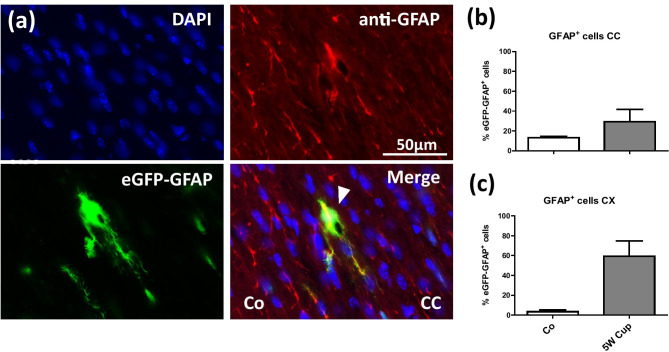
Immunohistochemical analysis of anti-GFAP (red) in eGFP-GFAP (green) transgenic mice. **(a)** Representative sections of anti-GFAP and eGFP-GFAP in the CC of control mice. The white arrow indicates the co-localization of anti-GFAP and eGFP-GFAP^+^. **(b)**, **(c)** The quantification of eGFP-GFAP^+^/anti-GFAP^+^ co-localization in the CC and CX, respectively. Three animals per groups; two independent experiments. Scale bar = 50 µm

Finally, we were interested, whether eGFP^+^ cells express as well GFAP, detected by immunohistochemistry. To this end, we processed brain sections from control and cuprizone-intoxicated transgenic mice for anti-GFAP immunofluorescence staining and quantified the co-localization of the signals. In the CC of control mice, 13.1% of all eGFP^+^ cells co-expressed GFAP whereas 29.2% of eGFP^+^ cells co-express GFAP in the CC of cuprizone-intoxicated mice. In the CX of control mice, 3.4% of all eGFP^+^ cells co-expressed GFAP whereas after cuprizone-intoxication 59.2% of eGFP^+^ cells co-express GFAP (see Fig. 5).

## Discussion

In this study, we were able to demonstrate that a significant number of cells labeled by the eGFP under the control of the human GFAP promoter express markers of the oligodendrocyte lineage such as OLIG2, NG2, or APC/CC1. The percentage of eGFP^+^ cells expressing such markers in the corpus callosum was low in control animals (~ 5% for anti-OLIG2 and anti-CC1; ~ 15% for anti-NG2) but significantly increased during the course of cuprizone-induced demyelination (~ 51% for anti-OLIG2, ~ 23% for anti-CC1, ~ 39% for anti-NG2). This increase was mainly observed in the white matter corpus callosum, but not in the grey matter cortex, albeit several studies were able to demonstrate that cuprizone induces demyelination in both areas (Clarner et al. 2012; Skripuletz et al. 2008).

Nolte and colleagues (Nolte et al. 2001), who developed and described this mouse strain for the first time, did not observe any co-localization with oligodendrocytes. In their studies, brain sections from eGFP transgenic mice were co-immunolabeled with the oligodendrocytic marker myelin-associated glycoprotein (MAG) and no co-localization was observed. In contrast to our current study, Nolte and colleagues investigated the cerebellum and striatum for the co-localization studies, whereas our regions of interest were the white matter tract corpus callosum as well as parts of the neocortex. While we observed a clear and frequent co-localization of eGFP and oligodendrocyte markers in the corpus callosum, such a co-localization was less frequently observed in other brain regions such as the cortex (see Fig. 3c) or the striatum (data not shown). This suggests that regional differences might exist with respect to the expression of oligodendrocyte marker proteins in the eGFP^+^ cells. Beyond, anti-MAG antibodies label both, the mature oligodendrocyte cell bodies, and the surrounding myelin. As already stated in the introduction section of this manuscript, when staining for oligodendrocytes with anti-MAG antibodies, the cell bodies might be masked by the abundant surrounding myelin, making it difficult to precisely delineate an oligodendrocyte cell body.

As demonstrated in Fig. 5, in both the white matter corpus callosum and the grey matter cortex, most eGFP^+^ cells do not express GFAP under control conditions, whereas in cuprizone intoxicated mice, expression overlap significantly increases. These results are in line with a previous study using the same mouse strain. There, it was shown that not all eGFP^+^ cells express GFAP and, vice versa, not all GFAP^+^ cells express eGFP (Nolte et al. 2001). In particular, in retinal or hypothalamic astrocytes, which normally show high GFAP immunoreactivity, eGFP fluorescence was either very low or even undetectable. On the other hand, eGFP-expressing cells with typical morphologies of gray matter protoplasmic astrocytes were poor in GFAP immunoreactivity as illustrated for some thalamic nuclei. As demonstrated in Fig. 2, anti-GFAP^+^ astrocytes are unevenly distributed throughout the adult brain. For example, numbers of GFAP-immunoreactive astrocytes are low in the grey matter cortex of control mice. Thus, GFAP-negative astrocytes predominate in the gray matter cortex (Ludwin et al. 1976). Due to their low GFAP expression levels, most grey matter astrocytes are, therefore, not visible which explains the relatively low co-localization of anti-GFAP and eGFP in the control mice. As one would expect, co-localization increases after cuprizone-induced demyelination since GFAP expression is now highly upregulated (see Fig. 5).

In a previous study it has been shown that anti-CC1 co-localizes with the oligodendrocyte marker protein NogoA but does not co-localize with the astrocytic marker GFAP after cuprizone-induced demyelination (Salinas Tejedor et al., 2015). It was, therefore, concluded that anti-CC1 antibodies do not label astrocytes in the cuprizone model. On the first view, these results appear to contradict our observation. However, when we co-labeled brain sections from cuprizone-intoxicated wild-type mice with anti-CC1 and anti-GFAP antibodies, we neither observed any clear co-localization (data not shown). As already shown by others (Ludwin et al. 1976), in many anti-GFAP^+^ cells, the perinuclear cytoplasm stains less strongly than the various cellular processes. In fact, in many astrocytes GFAP is not evenly distributed, but spares one site of the cell body (for example see Fig. 5 in (Zhan et al. 2020)). Co-localization studies are, thus, complicated in case perinuclear marker proteins are used such as anti-CC1 antibodies. Our results clearly show that at least in the applied mouse strain, a significant proportion of eGFP^+^ cells co-label with the applied anti-CC1 antibodies. However, one should keep in mind that the applied mouse strain in this study uses a human GFAP promoter to drive eGFP expression. Cre/loxP fate-mapping studies showed that the human, but not the mouse GFAP promoter, is active in oligodendrocyte progenitor cells (Casper and McCarthy 2006). It might, thus be that the expression of eGFP in cells of the oligodendrocyte lineage is an artefact of the used human GFAP promoter. Beyond, the possibility remains that our results are due to the absence of regulatory elements necessary for restricting eGFP expression to astrocytes.

A large number of investigators, including ourselves, have used the applied GFAP promoter to drive transgene expression in astrocytes. Results from these studies are routinely interpreted as due to the expression of the transgene in astrocytes. The current finding that the GFAP promoter drives transgene expression in cells of the oligodendrocyte lineage should be considered when interpreting results from such studies. In a previous study, our group has used this mouse strain to investigate which cells types express the Translocator Protein (TSPO), formerly known as the “peripheral benzodiazepine receptor,” in the cuprizone model (Nack et al. 2019). TSPO-radioligands are used in our days to visualize neuroinflammatory processes by Positron emission tomography (PET) imaging. While most studies suggest that TSPO is predominantly expressed by activated microglia cells and in consequence an increase in TSPO ligand binding is interpreted as microglia activation (Abourbeh et al. 2012; Airas et al. 2015; Blume et al. 2018; Klein et al. 2018; Mattner et al. 2013), various other cell types have been shown to express TSPO as well, among astrocytes (Mattner et al. 2011; Notter et al. 2018). In our studies, we were able to demonstrate a co-localization of anti-TSPO with eGFP, and this was interpreted as an astrocytic expression of TSPO. In light of the finding that eGFP is as well expressed by cells of the oligodendrocyte lineage, we might speculate that TSPO expression after cuprizone-induced demyelination is not just restricted to microglia and astrocytes (Nack et al. 2019), but as well oligodendrocytes might express the mitochondrial protein TSPO.

Since several years, two different lineage models dominate the research area of oligodendrocyte development. The glial-restricted precursor model proposes that glial cells (oligodendrocytes and astrocytes) arise from a common glia-restricted GFAP^+^ precursor cell and that glial-restricted precursor cells do not give rise to neurons. Alternatively, the motor neuron-oligodendrocyte precursor model proposes that oligodendrocytes and motor neurons share a common precursor cell, and that this precursor cell does not give rise to astrocytes. NG2 and OLIG2 are both expressed by oligodendrocyte progenitor cells. Considering the GFAP^+^ stem cell origin of many oligodendrocytes, the co-expression of eGFP and NG2 or OLIG2 is maybe not surprising. Indeed, there are prior reports of GFAP expression in cells of the early oligodendroglial lineage. For example, GFAP^+^/MBP^+^ cells have been described in fetal human and mouse spinal white matter (Choi and Kim 1984, 1985) and GFAP^+^/myelin oligodendrocyte-specific protein^+^ cells have been found in central white matter tracts of mice. In cultures, mixed phenotype glia were detected that were GFAP-positive and either MOSP-, MBP-, O1-, and O4-positive (Dyer et al. 2000). With respect to OLIG2 it has been shown that reporter-positive cells in *Olig2* reporter mice co-labeled with GFAP (around 5% after cortical stab wound injury; (Dimou et al. 2008)), and MBP-lacZ mice show a similar pattern of reporter/GFAP co-expression (Dyer et al. 2000).

Comparably, phenotypic overlaps have been reported in astroglia and oligodendroglia. GFAP-expressing progenitor cells can produce neurons and oligodendrocytes throughout the CNS (Casper and McCarthy 2006). Although the NG2 chondroitin sulphate proteoglycan is widely accepted as a marker for oligodendrocyte progenitor cells during development (Nishiyama et al. 1996; Polito and Reynolds 2005), these cells can transform into reactive astrocytes under pathological and cell culture conditions (Hall et al. 1996; Honsa et al. 2012; Leoni et al. 2009). Furthermore, it has been shown that astrocytes in the developing and adult rat optic nerve as well as in the adult rat grey matter, identified by their unambiguous ultrastructural features or by the commonly used GFAP^+^ criterion, widely express the NG2 proteoglycan (Alghamdi and Fern 2015). There is also convincing evidence for NG2/GFAP co-expression in astrocytes raised in culture conditions (Hirsch and Bähr 1999; Levine and Stallcup 1987).

During early development, neuroepithelial cells elongate and transform into radial glial cells. Neurons are the first wave of cells to emerge from radial glial cells of the neural tube followed by astrocytes and oligodendrocytes (Armati and Mathey 2010; Kriegstein and Alvarez-Buylla 2009). OLIG2 as a basic helix-loop-helix is expressed by neural stem cells and is present throughout the oligodendrocyte development to regulate oligodendrocyte lineage specification. However, the role of OLIG2 in astrocytes development has not been fully uncovered yet (Marshall et al. 2005). Previous studies reported that anti-OLIG2^+^ cells can generate astrocytes in a region specific manner. For example, OLIG2 stimulates astrocyte development in the dorsal pallium, but not in the basal forebrain (Ono et al. 2008). In addition, OLIG2 plays a crucial role in the development of astrocytes at early neonatal stages within the white matter; however, the expression of OLIG2 is progressively downregulated at late postnatal stages (Cai et al. 2007). Our results showed that eGFP-GFAP^+^ cells co-express OLIG2 and APC in the CC of cuprizone-intoxicated mice. In line with our results, Tatsumi and colleagues showed in the injured state that OLIG2 cell lineage preferentially differentiate into GFAP^+^ astrocytes (Tatsumi et al. 2008). One possible explanation for the co-localization of eGFP-GFAP^+^ cells with the applied oligodendrocyte markers is that during a stress situation, GFAP lineage cells show an interphase phenotype and by expressing oligodendrocyte markers (OLIG2, NG2, APC) adopt an oligodendrocytic phenotype (Mokhtarzadeh Khanghahi et al. 2018).

Although the exact fate and function of the eGFP-GFAP^+^ cells expressing markers of the oligodendrocyte lineage remains to be clarified in future studies, results of this study suggest that not all anti-CC1^+^ cells are mature oligodendrocytes, and a continuum might exist between activated astrocytes and oligodendrocytes (Ghasemi-Kasman et al. 2018; Mokhtarzadeh Khanghahi et al. 2018). Of note, activated astrocytes re-express, once stressed, marker proteins of their early developmental stages such as BLBP/FABP7 (Kipp et al. 2011a, 2011b) and might, thus, as well re-express progenitor markers such as NG2 and/or OLIG2. Most importantly, the current finding that the GFAP promoter drives transgene expression in cells of the oligodendrocyte lineage should be considered when interpreting results from co-localization studies.
